# IL-33 induces stronger responses in female mast cells and neutrophils: a role for the JNK pathway

**DOI:** 10.3389/fimmu.2026.1813530

**Published:** 2026-07-07

**Authors:** Sydney Ann Kee, Tania D. Maldonado, John M. Ching, Catherine Nelson, Zakaria Y. Hussain, An Z. Yao, Aditya Kotha, Hadi Hamze, Jason R. Burchett, John J. Ryan

**Affiliations:** 1 School of Life Sciences and Sustainability, Virginia Commonwealth University, Richmond, VA, United States; 2 Department of Microbiology and Immunology, Virginia Commonwealth University School of Medicine, Richmond, VA, United States; 3 Department of Cellular, Molecular, and Genetic Medicine, Virginia Commonwealth University School of Medicine, Richmond, VA, United States

**Keywords:** IL-33, inflammation, mast cell, neutrophil, sexual dimorphism

## Abstract

**Introduction:**

Allergic diseases exhibit considerable sex-based variations, with greater incidence of allergic asthma, atopic dermatitis, food allergy, and chronic urticaria among adult females. This female dominance may be explained by differences in inflammatory signaling.

**Methods:**

We used primary mouse mast cells, neutrophils, and eosinophils as well as two mouse models of IL-33-induced inflammation to measure male and female responses to IL-33.

**Results:**

We show that the allergy-associated cytokine IL-33 elicits stronger responses from female mast cells and neutrophils compared to males, both *in vitro* and *in vivo*. The greater female response was not due to increased expression or sensitivity of the IL-33 receptor in female cells. Instead, we noted female-selective activation of the JNK pathway in response to IL-33. Male and female mast cells both required the ERK, p38, NF-kB, and AP-1 pathways to produce inflammatory cytokines in response to IL-33. However, inhibiting the JNK pathway selectively reduced IL-33-mediated cytokine production in female mast cells. A mast cell-dependent model of IL-33-induced peritonitis showed that gonadectomy ablated sexual dimorphism in the neutrophil influx response. However, plasma IL-13 levels remained higher in females even among mice lacking sex organs.

**Discussion:**

These data show that IL-33 induces female-dominant mast cell and neutrophil responses, partly via female-selective JNK signaling.

## Introduction

Allergic diseases are common, affecting 1 in 4 adults and 1 in 5 children in the US (1). These inflammatory conditions include allergic asthma, allergic rhinitis, chronic urticaria, and food allergy. They share a Th2-biased inflammatory response that promotes IgE production and subsequent mast cell activation (2, 3). Mast cell-derived histamine, proteases, lipid mediators, and inflammatory cytokines directly cause many allergic symptoms by eliciting itch and vasodilation. These mediators also recruit eosinophils and other immune cells into the tissue that contribute to tissue damage and remodeling (4).

Mast cells respond to many stimuli in addition to IgE. Among the most clinically relevant is the cytokine IL-33, which also functions as a danger-associated molecular pattern (DAMP) (5, 6). IL-33 is constitutively produced by epithelial and endothelial cells, which store the precursor protein bound to DNA and release it upon cell damage. Processed, mature IL-33 binds to the IL1RL1 receptor, also known as suppression of tumorigenicity 2 (ST2). ST2 is prominently expressed by mast cells and group 2 innate lymphocytes (ILC2). It also activates eosinophils, basophils, neutrophils, and a subset of Th2 cells (7, 8).

An important aspect of allergic diseases is a sexual dimorphism that often exhibits increased prevalence and severity among females. For example, asthma affects 9.6% of adult females in the US, compared to 6.3% of males (1.5X difference) (9). Women are also more likely to experience severe symptoms and account for two-thirds of asthma-related deaths (9). Similar dimorphism is noted among US adults diagnosed with atopic dermatitis (1.5X more common in females), food allergy (1.9X more common in females), and chronic urticaria (2.1X more common in females). While the reasons for these disparities are incompletely understood, male-female differences often occur or shift during puberty, suggesting sex hormone effects. In support of this, some studies show that estrogen can play a pro-inflammatory role in asthma by promoting the release of histamine from mast cells and basophils, and enhancing the Th2 response (10–12). Conversely, testosterone is considered protective against inflammation. For example, it reduces the number of pulmonary ILC2 and the cytokines they produce (13, 14). Understanding how sexual dimorphism impacts allergic inflammation could lead to more effective, sex-specific treatments.

IL-33 signaling may contribute to sexual dimorphism in allergic disease. In support of this, Zhao et al. demonstrated that female mice challenged with IL-33 exhibited greater pulmonary ILC2 and eosinophil numbers than males (15). Mathä et al. similarly found greater ILC2 and eosinophil expansion after intranasal IL-33 administration in female mice (16). This group also demonstrated that purified female ILC2s mounted stronger IL-33 responses *in vitro* than male ILC2s, secreting at least 2-fold more IL-5 and IL-13. These studies prompted our interest in how IL-33 may affect other innate immune cells in allergic disease.

Using mouse bone marrow-derived mast cells (BMMCs), we found that female-derived cells produced higher concentrations of inflammatory cytokines and chemokines than male-derived cells when stimulated with IL-33. These effects could be linked at least partly to increased IL-33-mediated activation of c-Jun N-terminal kinase (JNK) in female mast cells. In addition to mast cells, neutrophils from female mice exhibited greater IL-33-induced migration *in vitro* than their male counterparts. In contrast, we found that bone marrow-derived eosinophils exhibited robust IL-33-induced cytokine production, with no sex differences. We further tested these effects *in vivo* and found that intraperitoneal IL-33 injection elicited greater neutrophil and eosinophil recruitment in females than males. Pre-puberty castration or ovariectomy yielded mice that exhibited no sex differences in IL-33-induced neutrophil recruitment, supporting a role for sex hormones in the dimorphic IL-33 response. Our study highlights the essential role that sex plays in IL-33 function, data that can help direct effective treatments for allergic diseases.

## Materials and methods

### Mice

Male and female C57BL/6J, BALB/cJ, C3H/HeJ, and *Kit^Wsh/Wsh^* and *Il1rl1^fl/fl^* mice were purchased from Jackson Laboratory, bred, and maintained under specific pathogen-free conditions at the VCU animal facilities. *Epx^Cre/+^* mice were the kind gift of Elizabeth Jacobsen (Mayo Clinic, Scottsdale AZ) and were bred to *Il1rl1^fl/fl^* mice to generate eosinophil-restricted *Epx^Cre/+^*; *Il1rl1^fl/fl^* mice. All animal use was approved by the Virginia Commonwealth University Institutional Animal Care and Use Committee.

### Mouse mast cell and eosinophil culture

To generate bone marrow-derived mast cells (BMMC), bone marrow was isolated from mouse femurs. Red blood cells were lysed with ACK lysis buffer (Quality Biological). Because phenol red is an estrogen receptor agonist (17), cells were plated in RPMI (Millipore-Sigma) containing phenol red (female cultures) or lacking it (male cultures) to mimic the *in vivo* environment more closely. The RPMI base ingredient concentrations were the same except for l-glutamine, which was added separately to achieve the same final concentration. RPMI ingredients can be found at these URLs: https://bit.ly/4fFwRYD and https://bit.ly/4dK95Jt. Complete (c)RPMI was made by supplementing RPMI 1640 with 10% fetal bovine serum (Sigma), 2 mM L-glutamine (Quality Biological), antibiotic solution containing 100 U/ml penicillin G, 100 μg/ml streptomycin sulfate and 250 μg/ml Amphotericin B (Avantor), 5 μg/ml ciprofloxacin HCL (Enzo Life Sciences), 200 μg/ml Fungin (InVivoGen), 1 mM sodium pyruvate (Fisher Scientific), and 1 mM HEPES buffer (Fisher Scientific). BMMC were differentiated in culture for 21 days in IL-3 (30 ng/ml; BioLegend). Cells were maintained in IL-3 and SCF (20 ng/ml each; BioLegend) after day 21 of culture, at which point the cells were ≥90% KIT/FcϵRI-positive and expressed ST2, as determined by flow cytometry analysis.

Peritoneal mast cells (PMCs) from female and male mice were expanded from peritoneal lavage cells. Lavage cells were harvested from naïve mice using phosphate-buffered saline (PBS) with 5mM EDTA, then centrifuged at 400 x g for 5 minutes at 4°C. Red blood cells were lysed with ACK lysis buffer and the cells were then cultured for 10–14 days at 600,000 cells/ml in cRPMI described above, containing IL-3 (20 ng/ml) and SCF (50 ng/ml). Cells from male mice were cultured in media lacking phenol red, as described for BMMC. PMC phenotype was confirmed by staining for cell-surface KIT, FcϵRI, and ST2 expression, as determined by flow cytometry.

Bone marrow-derived eosinophils were differentiated by culture in RPMI supplemented as described for BMMC with the following changes: 20% FBS (Sigma-Aldrich), 25 mM HEPES (Cytiva), 55 μM 2-mercaptoethanol (Sigma-Aldrich), non-essential amino acids (Gibco) and Glutamax (Thermo Fisher). As with BMMC, female eosinophils were cultured in RPMI containing phenol red, while male cells were cultured in RPMI without phenol red. The media was supplemented with SCF and FLT3-ligand (100 ng/ml each; BioLegend) for days 1-4, followed by IL-5 (10 ng/ml; BioLegend) for days 5-10. Eosinophil phenotype was confirmed by cell surface expression as CDllb^low^ (BioLegend, clone M1/70) and SIGLEC-F^high^ (BioLegend, clone S17007L), as determined by flow cytometry.

### Flow cytometry

Surface staining was performed using cells at 1x10^6^ cells/ml in 500 μL PBS/3% fetal bovine serum at 4 °C for 30 minutes. Flow cytometry data were acquired using a FACSCelesta (Becton Dickinson) and analyzed by FACS Diva. Cells were gated on high FSC and SSC to exclude dead/dying cells, and FSC-H/FSC-A to exclude doublets. Further gates were determined using isotype and/or fluorescence minus-one (FMO) controls. Antibodies used included anti-Ly6C-FITC (BD Biosciences clone AL-21). The following antibody conjugates were purchased from BioLegend: anti-CD45-APC (clone 30-F11), anti-Ly6G-PE (clone HK1.4), anti-CD11b-PE (clone M1/70), anti-KIT-PE (clone 2B8), anti-ST2-BV421 (clone DIH4), and anti-FcϵRI-APC (clone MAR-1).

### Supernatant cytokine measurement

BMMC and eosinophils were plated at 1x10^6^ cells/ml in the media described above and activated for 16 hours with IL-33 at concentrations described in figure legends. Culture supernatants were analyzed by ELISA using the manufacturer’s instructions to detect mouse IL-6, MCP-1, and TNF-α (BioLegend) or mouse IL-13 and MIP-1α (Peprotech). ELISA data was acquired using a Multiskan FC Type 357 plate reader (Thermo Scientific).

### Western blot analysis

C57BL/6J BMMC (4x10^6^ cells) were plated in 1 ml of phenol red-containing cRPMI for females and cRPMI without phenol red for males, without growth factors, in a 24-well plate, and incubated for 5 hours at 37 °C with 5% CO_2_. After incubation, IL-33 (10 ng/ml) was added to stimulate the cells. Ice-cold PBS was added after 0, 10, or 30 minutes. Cells were then collected, centrifuged, and the media aspirated. To the cell pellets, 40 µL of RIPA lysis buffer and extraction buffer (Thermo Fisher) containing Halt protease and phosphatase inhibitor cocktail (Thermo Scientific) and 0.5 M EDTA (Thermo Scientific) was added, and the mixture was transferred to microcentrifuge tubes. The tubes were vortexed every 5 minutes for 1 hour, and lysates were collected for Pierce BCA protein assay (Thermo Scientific). Lysates were loaded into SDS buffer containing 2-mercaptoethanol and heated to 95 °C for 5 minutes. Equal amounts of protein (25 μg) from each sample were loaded and separated on a 4-20% SDS-polyacrylamide gel (BioRad) with a Precision Plus Protein Dual Color Standards ladder (BioRad). Once the gels were complete, they were transferred to a nitrocellulose membrane, and primary antibodies from Cell Signaling Technology (Danvers, MA) were applied overnight at 4°C. These antibodies recognized: Phospho (P)-SAPK/JNK (T183/Y185) (clone G9), SAPK/JNK (rabbit Ab, catalog #9252), P-p44/42 MAPK (T202/Y204) (rabbit Ab, catalog #9101), p44/42 MAPK (ERK 1/2) mouse mAb (clone L34F12), P-p38 MAPK (T180/Y182) mouse mAb (clone 28B10), p38 MAPK rabbit Ab (catalog #9212), P-NF-κB p65 (S536) rabbit Ab (catalog #3033S), NF-κB p65 (S536) mouse mAb (clone L8F6). Primary antibodies were detected using anti-mouse IgG (H+L)-680 (catalog #5470) and anti-rabbit IgG (H+L)-800 (catalog #5151) (Cell Signaling) at 1:15,000 for 1 hour at room temperature, then visualized using an Odyssey CLx imager (LI-COR).

### Arachidonic acid metabolites

Female and male C57BL/6 BMMC were plated at 2x10^6^ cells per sample in 2 ml of cRPMI (containing phenol red for female cultures; lacking phenol red for male cultures) without growth factors in a 24-well plate. After one hour, IL-3 and SCF were added (10ng/ml each), and BMMC were stimulated with 10 ng/ml of IL-33. One hour after stimulation, supernatants were collected, centrifuged at 440 x g for 5 minutes at 4°C to remove cells, and supernatants were stored at -80 °C. Supernatants were analyzed by the VCU Lipidomics and Metabolomics Shared Resource Facility using mass spectrometry. Samples were extracted using a modified Blight-Dyer method followed by the requested quantitative analysis. For targeted metabolites, internal standards and methods were used to yield data on species of interest within water-soluble classes/pathways. In addition, quantification used two distinct identifiers for each analyte: structure-specific fragmentation and UHPLC retention times. The remaining supernatants were collected after 16 hours and used to confirm sexual dimorphism by measuring IL-6 secretion via ELISA.

### Signal transduction inhibitors

The following signal transduction inhibitors were purchased from MedChemExpress: the ERK inhibitor SCH772984; the JNK inhibitor JNK-IN-8, the p38 inhibitor ralimetinib dimesylate, the NF-κB inhibitor bay11-7082, and the AP-1 inhibitor (6E)-SR 11302. All inhibitors were dissolved in dimethylsulfoxide, which was used as the vehicle control. Inhibitors were added 60 minutes before IL-33.

### Mouse neutrophil culture and migration

Bone marrow from the femurs of 10 C57BL/6J mice was used to purify neutrophils. Red blood cells were lysed with ACK lysis buffer. Cells in PBS were placed on top of Histopaque 1077 (Sigma Aldrich), which was stacked on top of Histopaque 1119 (Sigma Aldrich) and centrifuged at 834 x g at 20 °C for 30 minutes without braking. Cells were collected at the interface of the Histopaque layers, then washed twice with PBS (440 x g at 20 °C for 5 minutes) and plated in RPMI with 10% FBS (Sigma-Aldrich) supplemented with 100 μg/ml penicillin, 200 μg/ml streptomycin and 250 μg/ml Amphotericin B. Neutrophil purity was confirmed by flow cytometric analysis for expression of CD45, CD11b, and Ly6G.

For migration, 1x10^6^ cells in 700 µL RPMI containing 1% fetal bovine serum, penicillin, streptomycin, and Amphotericin B (same concentrations as above) were placed in the top chamber of 5 μm-diameter Transwells (Corning) for four hours. The media used for female cells contained phenol red; media used for male cells lacked phenol red. Migration stimuli described in the figure legends was added to the bottom chamber. Migrating cells were collected from the bottom chamber in 3 x 200 μl samples. Propidium iodide dye exclusion was used to count live cells by flow cytometry for 30 seconds/sample. Fold migration was calculated by dividing the number of live cells in the experimental samples by the average of the number of live cells in control samples (media alone).

### IL-33-mediated neutrophilic peritonitis

Mice were injected intraperitoneally (i.p.) with 1 μg IL-33 (Biolegend) dissolved in PBS or with PBS alone. Four hours after IL-33 or PBS injection, peritoneal lavage cells were collected in PBS with 5 mM EDTA. Peritoneal cells were blocked with anti-CD16/CD32 clone 2.4G2 and stained at 4 °C for 30 minutes with anti-CD45, Ly6G, and Ly6C. Neutrophils were identified by flow cytometry as CD45^+^, Ly6G^high^, Ly6C-negative cells after doublet discrimination. Peripheral blood was also collected via cardiac puncture, placed in microcentrifuge tubes containing 10 μl 0.5M EDTA, and centrifuged at 400xg for 5 minutes at 4 °C. Liquid blood plasma was stored at -80 °C.

### IL-33-mediated eosinophilic peritonitis model

Mice were injected i.p. with 0.5 μg IL-33 (Biolegend) dissolved in PBS or with PBS alone daily for 4 days. Four hours after the last injection, peritoneal lavage cells and peripheral blood plasma were collected as described above. Peritoneal cells were blocked with anti-CD16/CD32 clone 2.4G2 and stained at 4 °C for 30 minutes with anti-CD45, F4/80, Siglec-F, CD11b, and Ly6-G. Eosinophils were identified by flow cytometry as CD45+, Siglec-F+, CD11b^low^, Ly6G^low^ cells after doublet discrimination.

### Statistical analysis

GraphPad Prism software version 10 was used to calculate p-values. Data in each figure are expressed as mean ± standard error of mean (SEM) with statistical significance as follows: *p< 0.05, **p< 0.01, ***p< 0.001, ****p<0.0001. The abbreviation “n.s.” means non-significant. Biological replicates for *in vitro* experiments were cell populations cultured from separate mice. Biological replicates for *in vivo* experiments were individual mice. Technical replicates were conducted for all experiments, consisting of duplicate or triplicate samples. Unless otherwise stated, comparisons between two groups were performed using Student’s t-test; for three or more groups, ANOVA was used with the software-suggested default *post-hoc* test for multiple comparisons, as stated in figure legends.

## Results

### Phenol red is required for female mast cell expansion but hinders male mast cell expansion and function

Before determining if mast cells exhibit sexually dimorphic responses to IL-33, we first examined cell culture conditions for deriving BMMC. Phenol red binds the estrogen receptor (ER) with a Kd of 20 μM (17). Since phenol red is present in RPMI base media at 15 μM, it likely acts as an estrogen signal. In standard culture media, phenol red can induce ER-dependent MCF-7 cell line growth and prolactin release from male pituitary cells (17, 18). Hence, we tested the impact of phenol red on BMMC culture.

Male and female bone marrow cells were cultured with 30 ng/ml IL-3 in standard phenol red-containing cRPMI or in matched phenol red-free cRPMI for 31 days and counted. Male cells showed >2-fold greater expansion in media lacking phenol red. In contrast, female bone marrow cells exhibited 3X greater expansion in media containing phenol red (**Supplementary Figure 1A**). Thus, phenol red, possibly due to its estrogenic effects, appears to be required for optimal expansion of female mast cells but inhibits male mast cell proliferation.

In addition to cell number, we also tested IL-33-induced IL-6 production in these cultures. Phenol red did not affect IL-6 secretion by female mast cells (**Supplementary Figure 1B**). However, male BMMC cultured in media containing phenol red produced 70% less IL-6 than matched cultures lacking phenol red. More importantly, we noted that male cells produced less IL-6 than female cells under either culture condition. We therefore continued our studies with female BMMC cultured in media containing phenol red and male cells in media lacking this dye.

### IL-33-induced mast cell cytokine and arachidonic acid production exhibits stronger responses in females

**Figure 1:**
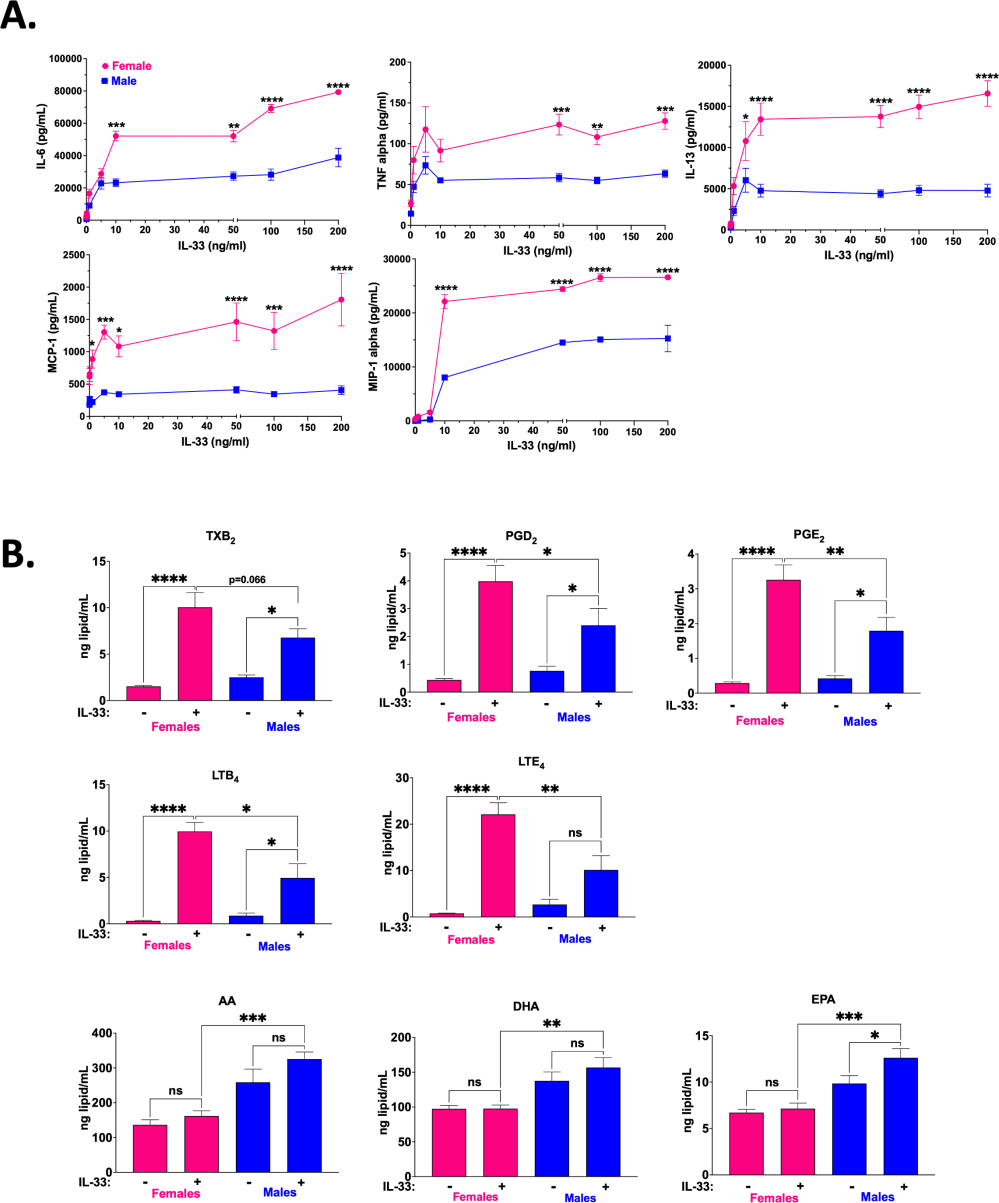
IL-33 induces greater cytokine and arachidonic acid metabolites production from female than from male mast cells. **(A)** Male and female BMMC were stimulated for 16 hours with IL-33 at the indicated concentrations and supernatants were analyzed by ELISA to measure cytokine and chemokine concentrations. **(B)** Male and female BMMC were activated +/- IL-33 (10 ng/ml) for one hour, and culture supernatants were analyzed for arachidonic acid metabolite concentrations by mass spectroscopy. Data shown are from 3 male and 3 female BMMC cultures analyzed in duplicate in 2 independent experiments **(A, B)**. P-values were calculated in **(A)** using one-way ANOVA with Šídák’s multiple comparisons test. The p-value when comparing unstimulated male and female samples was 0.38. P-values were calculated in **(B)** using one-way ANOVA with Barlett’s posthoc test. *p< 0.05, **p< 0.01, ***p< 0.001, ****p<0.0001.

To determine if there are sex differences in mast cell responses to IL-33, male and female BMMC were stimulated with varying IL-33 concentrations for 16 hours. Female BMMC released larger amounts of cytokines when stimulated with IL-33 at concentrations greater than 10 ng/ml, although TNF-α production was not significantly different below an IL-33 concentration of 50 ng/ml (**Figure 1A**). Compared to males, female BMMC produced approximately 2-fold more IL-6, TNF-α, and MIP-1α, and >3-fold more IL-13 and MCP-1. Interestingly, these differences were not linked to IL-33 sensitivity. For example, the EC_50_ for IL-33-induced IL-6 secretion was 6.25 ± 0.53 ng/ml (mean ± SEM) for female BMMC and 4.13 ± 0.85 ng/ml for males (p=.053 by t-Test). These data indicated that female mast cells are not more sensitive to IL-33 but mount a response of greater magnitude than male cells.

To determine if differences in genetic background influence this apparent sexual dimorphism, we compared IL-33 dose-responses using C3H/HeJ and BALB/cJ BMMC (**Supplementary Figure 2**). We observed a similar trend to that seen with the C57BL/6J BMMC, with female cells consistently producing greater concentrations of cytokines and chemokines.

In addition to cytokines, IL-33 stimulates the production of arachidonic acid metabolites. We examined differences in arachidonic acid metabolites secreted by female and male BMMC after IL-33 stimulation for one hour (**Figure 1B**). We found that female cells produced approximately 2-fold greater amounts of the inflammatory lipids thromboxane B_2_, prostaglandins PGD_2_ and PGE_2_, and leukotrienes LTB_4_ and LTE_4_ than male mast cells. Interestingly, male cells were not deficient in arachidonic acid and contained slightly greater amounts of the anti-inflammatory metabolites docosahexaenoic acid (DHA) and eicosapentaenoic acid (EPA), although these lipid levels did not change with IL-33 stimulation. We conclude that female mast cells mount a stronger inflammatory lipid response to IL-33 than male mast cells in these culture conditions.

**Figure 2:**
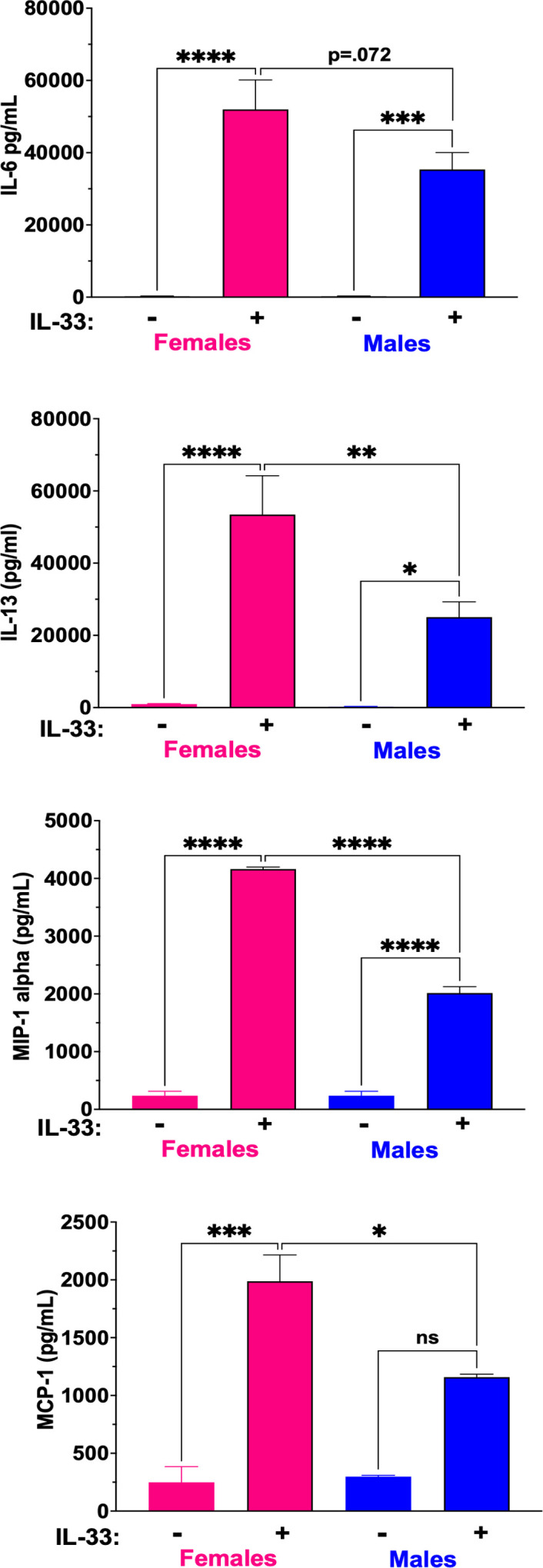
Peritoneal mast cells show greater IL-33-induced cytokine production from female than from male mice. Female and male PMCs were stimulated with IL-33 (10 ng/ml) for 16 hours. Supernatants were collected, and cytokine ELISAs were performed. The data shown are from 3 male and 3 female PMC samples analyzed in duplicate in 2 independent experiments. P-values were determined by one-way ANOVA with Barlett’s posthoc test. *p< 0.05, **p< 0.01, ***p< 0.001, ****p<0.0001.

We also examined whether there is sexual dimorphism among peritoneal mast cells (PMC), which differentiate *in vivo*. Much like BMMC, female PMC produced higher levels of cytokines and chemokines than their male counterparts (**Figure 2**). Thus, our data are consistent using BMMC from multiple genetic backgrounds and were corroborated with PMC.

### The female-dominant response to IL-33 is linked to increased JNK activation

The greater magnitude of IL-33 responsiveness in female BMMC might be explained by increased expression of the cognate receptor, ST2. In addition, ST2 function can be amplified by KIT signaling (19). Therefore, we measured cell surface expression of ST2 and KIT by flow cytometry. Male and female BMMC were uniformly positive for ST2 and KIT expression and mean fluorescent intensity (MFI) analyses showed no difference in expression. We also measured FcϵRI and similarly found no differences (**Supplementary Figures 3A, B**). We also found that the fraction and total number of mast cells in the peritoneal cavity did not differ between males and female mice (**Supplementary Figure 3C**). Similarly, PMCs also showed no sex differences in expression of KIT, FcϵRI, or ST2 (**Supplementary Figure 4**). Therefore, the dominant IL-33 response of female mast cells is not caused by increased receptor expression.

**Figure 3:**
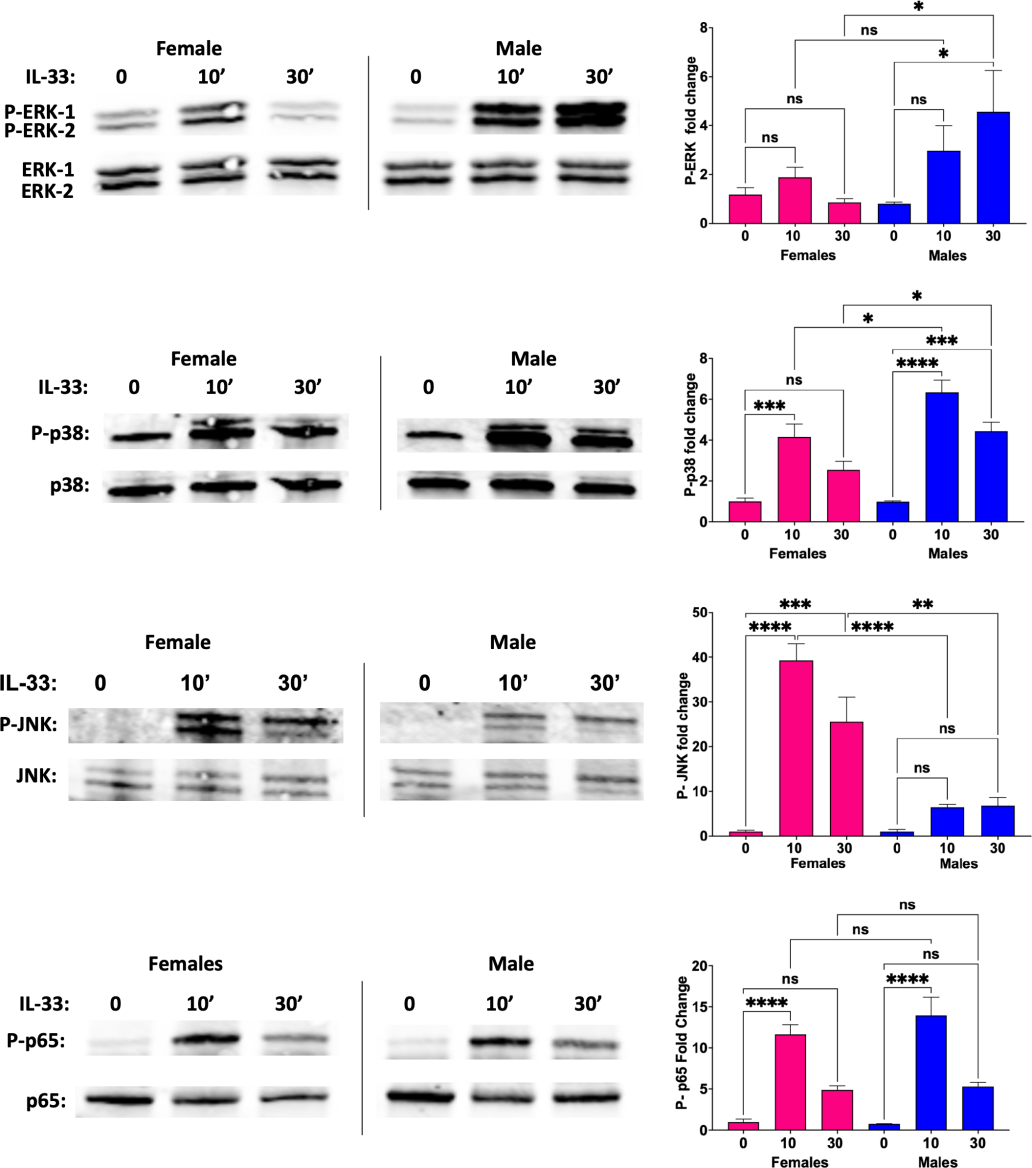
IL-33 induces JNK activation selectively in female mast cells. Female and male BMMCs were starved for 5 hours before being stimulated with IL-33 (10 ng/ml) for 10 or 30 minutes. Lysates were analyzed by Western blot to measure phosphorylation of the indicated proteins. Representative blots are shown on the left and matched bar charts show the mean +/- SEM of phospho-protein/total protein ratio. The results shown are representative of 3 female and 3 male independent cultures, with 2 experiments done in duplicate. 1-way ANOVA determined P values with Barlett’s posthoc test. *p< 0.05, **p< 0.01, ***p< 0.001, ****p<0.0001.

We next determined if there are differences in IL-33 signal transduction among female and male BMMC. We performed Western blots and calculated fold changes in the activation of MAP kinases and the NF-κB p65 subunit (**Figure 3**). This revealed several sex-based differences. IL-33 induced ERK phosphorylation in male BMMC, while the female response did not reach significance. Male mast cells also exhibited 1.5-fold greater IL-33-induced p38 activation than females. Perhaps most strikingly, female BMMC showed nearly 40-fold JNK phosphorylation in response to IL-33, while male mast cells did not activate JNK. Finally, we found no difference in p65 phosphorylation between male and female cells. These data point to JNK as a possibly important difference in the female response to IL-33.

**Figure 4:**
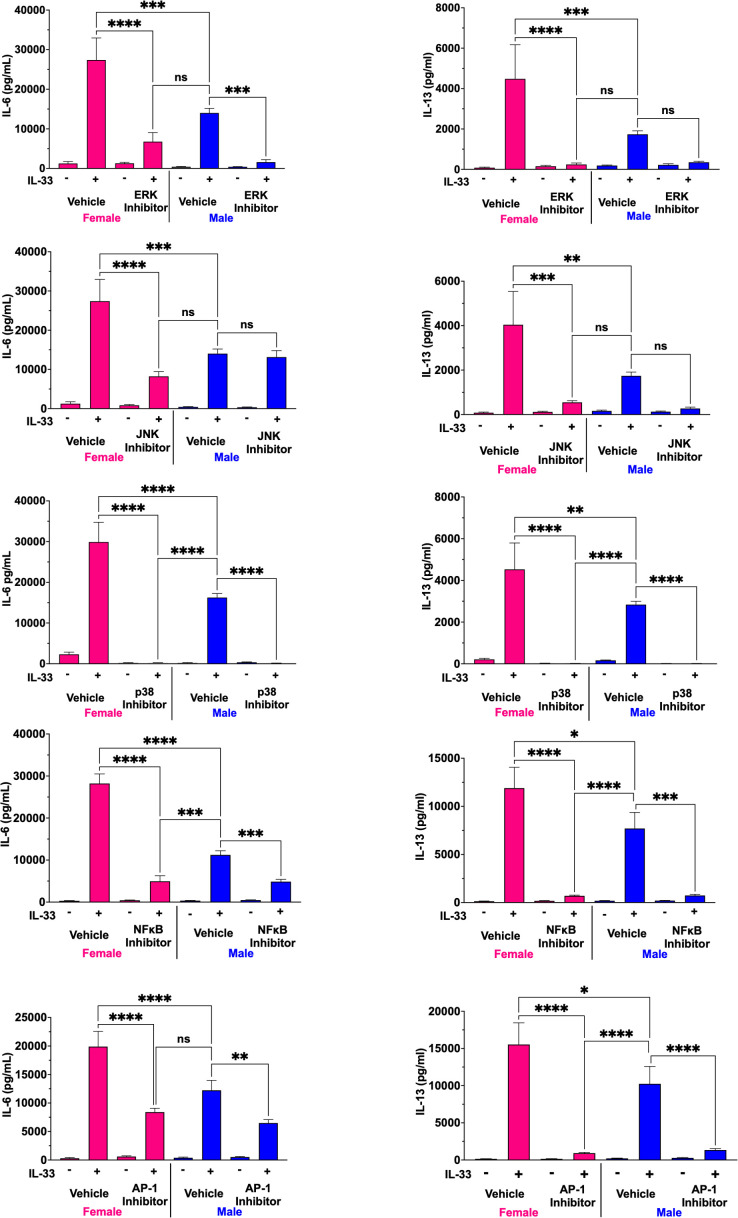
JNK inhibition suppresses cytokine production selectively in female mast cells. BMMC were pretreated with inhibitors (ERK: SCH772984; JNK: JNK-IN-8; p38: Ralimetinib dimesylate; NF-κB: bay11-7082; AP-1: (6E)SR 11302) for one hour. Bay11–7082 was used at 5 μM final concentration; all other inhibitors were used at 10 μM. Cells were then activated with 10 ng/ml of IL-33 for 16 hours. Supernatants were collected and analyzed via ELISAs. Data are shown as the SEM of two independent experiments, each using 3 male and 3 female BMMC populations analyzed in triplicate. P values were determined by 1-way ANOVA with Šídák’s posthoc test. *p< 0.05, **p< 0.01, ***p< 0.001, ****p<0.0001.

To assess the functional relevance of these differences, we determined if inhibiting these pathways reduces IL-33-induced cytokine production (**Figure 4**). Importantly, under the conditions tested, none of the inhibitors affected cell viability, as measured by cell imaging on the LUNA II cell counter (data not shown). Greater IL-33 responses were consistently noted among females cells, consistent with the data shown in **Figure 1**. ERK or p38 inhibition significantly reduced IL-6 and IL-13 production by both male and female BMMC, indicating these pathways are critical for IL-33 responsiveness in both sexes. In contrast, JNK inhibition selectively decreased IL-6 and IL-13 production by female BMMC, with no significant effect on male BMMC. In the presence of a JNK inhibitor, IL-33-activated female BMMC produced cytokines at levels similar to male BMMC cultured without the inhibitor. These data agree with the phospho-JNK Western blot result and suggest that female-specific JNK activation contributes to greater IL-33-induced cytokine production relative to male cells. We also tested the effects of inhibiting NF-κB or AP-1, transcription factors lying downstream of the MAPK cascade and activated by ST2 signaling (20). Despite the variations in MAPK signaling found with Western blotting, blocking NF-κB or AP-1 greatly reduced IL-33-induced IL-6 and IL-13 secretion by male or female mast cells. We conclude that of the pathways examined, female-selective JNK activation is the most likely contributor to the sexually dimorphic mast cell response to IL-33.

### Female-dominant IL-33 effects are consistent *in vivo* and extend to neutrophils

**Figure 5:**
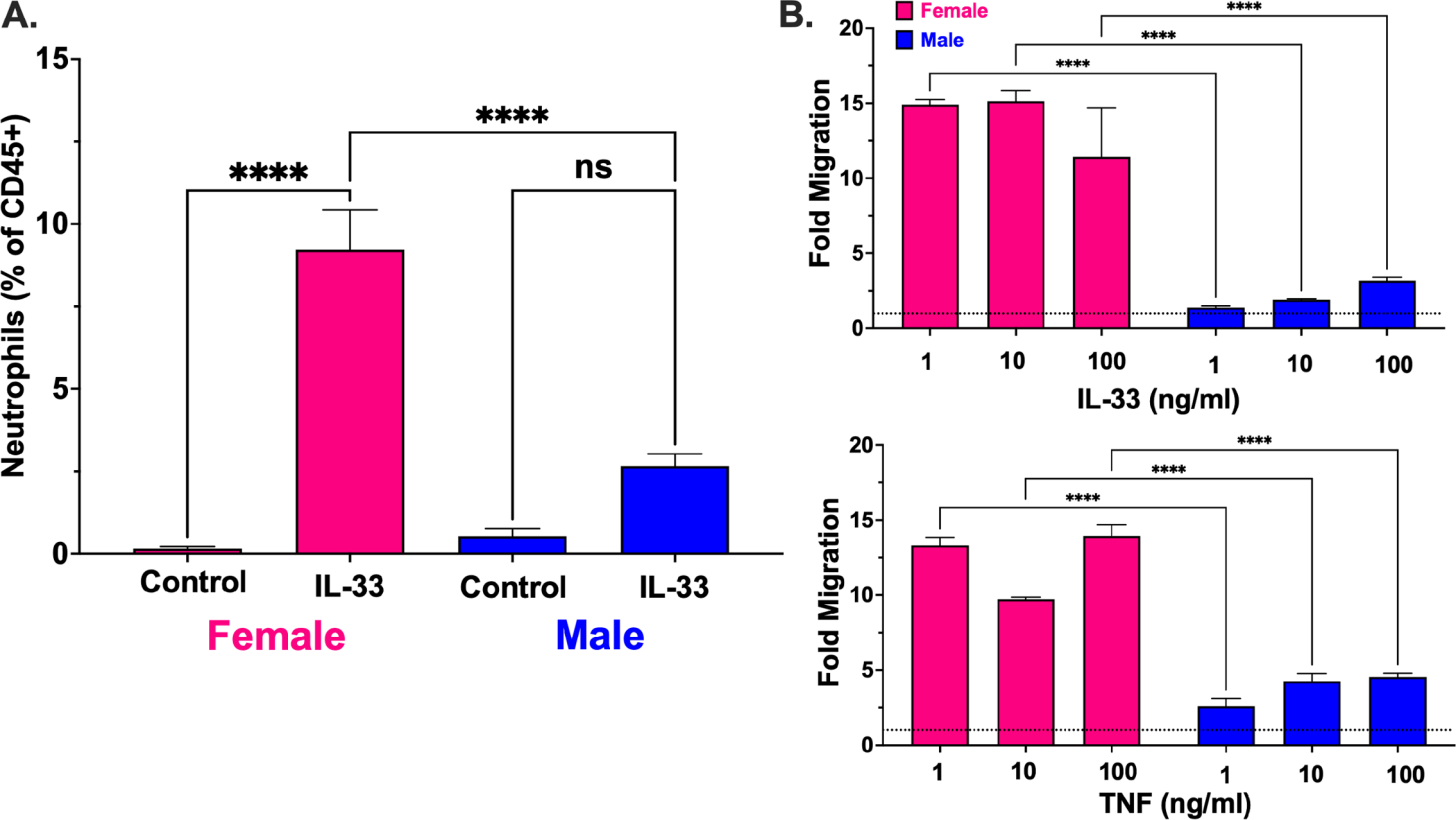
*In vivo* and *in vitro* models show IL-33 induces neutrophil migration selectively in females. **(A)** Mice received PBS or 1μg IL-33 injection i.p. and peritoneal lavage cells were harvested 4 hours later. Neutrophils were measured by flow cytometry in duplicate samples. n=10 females and 7 males from 2 experiments. **(B)** Neutrophils were isolated from naïve male and female C57BL/6J mouse bone marrow. Migration toward the indicated concentrations of TNF-α and IL-33 was measured using flow cytometry. The dotted line indicates 1-fold (control) migration in media alone. Data shown are from n=3 females and n=6 males analyzed in triplicate samples. P values were determined by 1-way ANOVA with Tukey’s posthoc test. ****p<0.0001.

To determine if sex-dependent IL-33 mast cell function is consistent *in vivo*, we tested a model of IL-33-induced peritonitis. Enoksson and coworkers showed that a single i.p. injection of IL-33 results in a neutrophil influx that requires mast cell-derived TNF-α secretion (21). Using this mast cell-dependent model, we injected male and female mice with IL-33 and measured neutrophils in peritoneal lavage after four hours. Female mice demonstrated a 3-fold greater neutrophil influx than males (**Figure 5A**). Because a standard amount of IL-33 (1 μg) was injected in this model, we next confirmed that the greater response in females was not due to weight differences. When using IL-33 at 50 ng/g (e.g., 1μg in a 20g animal), we again noted a >3X greater neutrophil influx in females, as judged by both percentage and number of cells (**Supplementary Figure 5**).

Neutrophil influx in this model requires mast cell-derived TNF-α (21). However, the stronger female response could also be due to a greater neutrophil response to IL-33 or TNF-α, separate from or in addition to the mast cell response. To test this, we measured neutrophil migration to IL-33 and TNF-α. Primary neutrophils from female mice exhibited approximately 3-fold greater migration to either stimulus compared to male neutrophils (**Figure 5B**). This was not due to differences in neutrophil expression of ST2, or TNF receptors 1 or 2, which were detectable on a small percentage of bone marrow neutrophils (**Supplementary Figure 6**). The difference in fold migration was also not due to a lower baseline migration in the reference (unstimulated) female group, which averaged 75 cells, compared to 55 cells in the male counterpart. Thus, neutrophils exhibited sex-dependence in response to IL-33 and TNF-α stimulation. These data support the hypothesis that sexual dimorphism in the IL-33 response is consistent in mast cells and neutrophils.

### IL-33-induced eosinophilic peritonitis also exhibits stronger responses in females than in males

**Figure 6:**
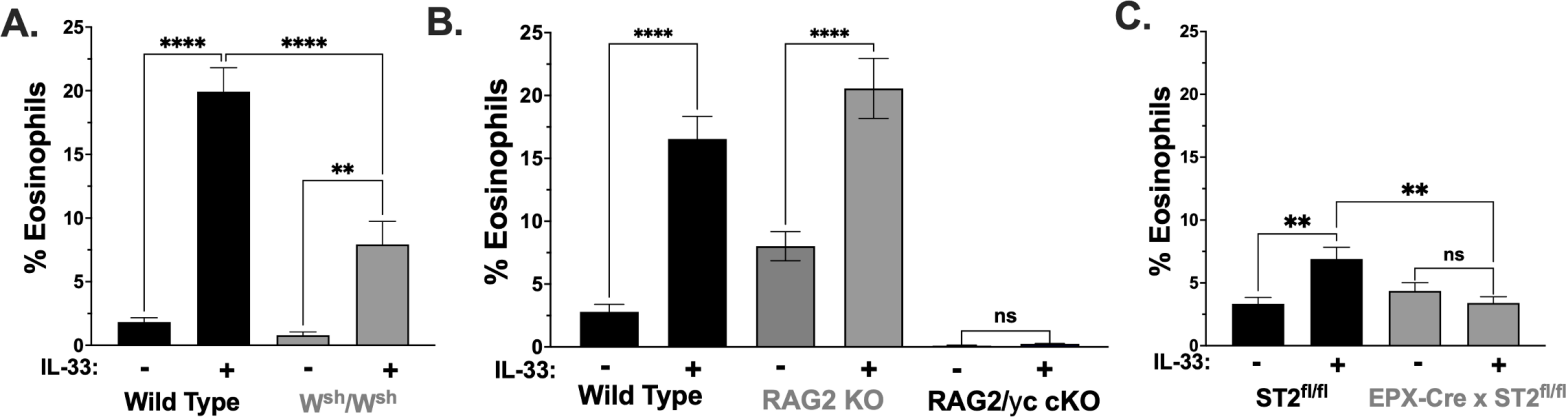
IL-33-mediated eosinophilic peritonitis demonstrates a pro-inflammatory role for mast cells, ILC, and ST2 expression on eosinophils. Female mice from (A) Mast cell-deficient, (B) lymphocyte- and ILC-deficient or (C) eosinophil-restricted ST2-deficient genetic strains and controls were injected with PBS or IL-33 daily for 4 days, and peritoneal lavage eosinophils were measured by flow cytometry. N=4 mice/group analyzed in triplicate. Cells were stained with CD11b-PE, Siglec-F-BV, and ST2-APC. Eosinophils were identified via flow cytometry. Total N = 11 *Il1rl1^fl/fl^* and N = 12 *Epx^Cre/+^*;*Il1rl1^fl/fl^*. Analyzed in triplicate. Significance was assessed using ordinary one-way ANOVA with Šídák’s posthoc test. **p< 0.01, ****p< 0.0001.

Because eosinophils also play a critical role in allergic diseases, we next tested an *in vivo* model of IL-33-induced eosinophilic inflammation, which employs four daily IL-33 i.p. injections. While the model has been established, the cellular requirements for eosinophil recruitment have not been described (22). To determine if eosinophilic recruitment was mast cell-dependent, we compared female C57BL/6J mice with female mast cell-deficient W^sh^/W^sh^ mice. This revealed a significant, albeit not complete, decrease in peritoneal eosinophilia, supporting a role for mast cells (**Figure 6A**). We then compared female RAG2/γc double KO mice to RAG2 KO mice and controls. The double KO mice have the lymphocyte deficiency found in RAG2 KO and also lack innate lymphoid cells (ILCs) (23, 24). This comparison showed that mature lymphocytes are not necessary for IL-33-mediated eosinophil recruitment, as RAG2 KO and C57BL/6J controls had no differences. In contrast, RAG2/γc double KO had a complete absence of eosinophil recruitment, supporting the hypothesis that ILC are essential for IL-33 effects (**Figure 6B**). Finally, we bred *Il1rl1*-^fl/fl^ to *Epx^Cre/+^* mice to determine if eosinophils must express the ST2 receptor to respond to IL-33 in this model. As shown in **Figure 6C**, mice lacking ST2 expression on eosinophils exhibited no eosinophil influx after IL-33 challenge, while floxed control mice did. This was not due to decreased eosinophil numbers in the *Epx^Cre/+^*; *Il1rl1^fl/fl^* mice. In fact, *Epx^Cre/+^*; *Il1rl1^fl/fl^* mice had increased eosinophil numbers in the bone marrow and no change in splenic eosinophil counts (**Supplementary Figure 7**). Collectively, these data indicate that IL-33-induced peritoneal eosinophilia requires ILC and mast cell activation and that IL-33 also acts directly on eosinophils.

**Figure 7:**
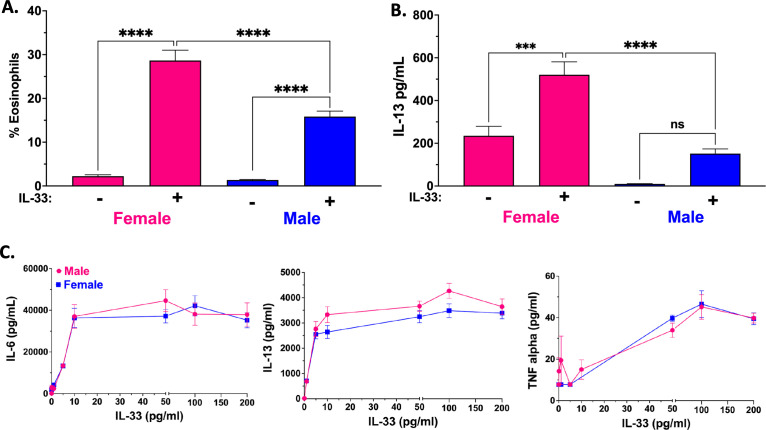
IL-33 injection induces greater eosinophil migration in females, while IL-33 stimulation *in vitro* shows no sex differences in IL-33-induced eosinophil cytokine production. C57BL/6J mice were injected with IL-33 daily for 4 days and peritoneal eosinophils **(A)** and plasma IL-13 **(B)** were measured on day 4. Data from 8 mice analyzed in duplicate in **(A)** and 4–6 plasma samples per sex, analyzed in duplicate in **(B)**; p-values from ANOVA with Tukey’s test. **(C)** Eosinophils cultured from bone marrow cells were stimulated with varying doses of IL-33 for 16 hours. ELISAs were used to analyze cell supernatants. n=6 male and 6 female eosinophil populations/group analyzed in duplicate in each of 2 experiments. Significance was assessed using one-way ANOVA with Šídák’s posthoc test. ***p< 0.001, ****p<0.0001.

We used this model to compare male and female C57BL/6J mice. As with the neutrophilic peritonitis model, males exhibited 50% less eosinophil recruitment than females (**Figure 7A**). We also noted that IL-33 induced significantly more plasma IL-13 in females than in males. In fact, males showed no significant IL-13 production in this model (**Figure 7B**). Therefore, we conclude that both neutrophilic and eosinophilic infiltration induced by IL-33 exhibit female-dominant sexual dimorphism.

Although our data show that IL-33-induced eosinophil migration requires that eosinophils express ST2, IL-33 itself may not be the migration stimulus. In fact, we measured migration of cultured eosinophils *in vitro*, and found no migration towards IL-33 in Transwell assays (data not shown), similar to the findings of others (25, 26). However, male and female eosinophils mounted a robust cytokine response to IL-33, producing IL-6, IL-13, and TNF-α (**Figure 7C**). Unlike mast cells, eosinophils from male and female mice showed nearly identical responses to IL-33, indicating no sexual dimorphism for IL-33-induced cytokine production. We conclude that while eosinophil ST2 function is required for IL-33-induced migration *in vivo*, IL-33 is likely not eliciting this response directly. Further, our data find no sexual dimorphism in the direct effects of IL-33 on eosinophils. Thus, sexually dimorphic IL-33 effects may be cell lineage-dependent.

### Sex hormones play a critical role in IL-33-induced neutrophilia

**Figure 8:**
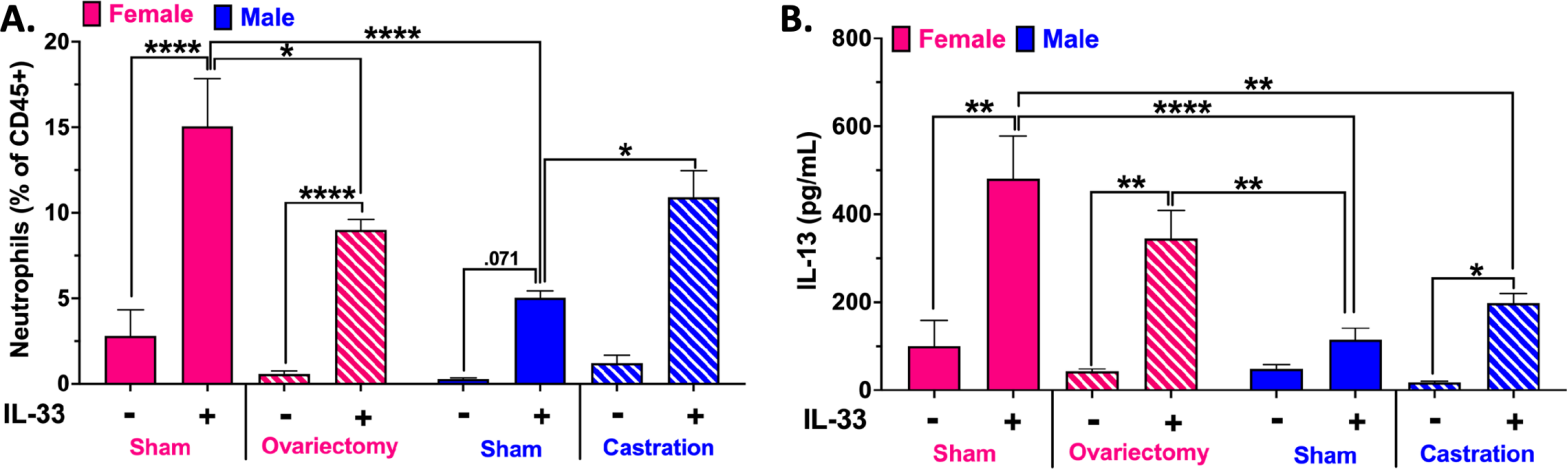
Female dominance in IL-33-mediated neutrophilic peritonitis is abrogated by removing sex organs before puberty. Mice received a 1 μg IL-33 injection i.p. four hours before peritoneal lavage was collected. **(A)** Neutrophils were identified by flow cytometry as CD45+ cells Ly6G^hi^ Ly6C^low^. **(B)** Plasma IL-13 was measured by ELISA. Data are from n=4–5 mice per group. Samples were analyzed in duplicate. Statistics were obtained using one-way ANOVA with Tukey’s posthoc test. *p< 0.05, **p< 0.01, ****p<0.0001.

In the context of immunity, estrogens are generally pro-inflammatory, while androgens are generally anti-inflammatory (27). To determine the effects of sex hormones on IL-33 signaling *in vivo*, we used spayed females and neutered males in the neutrophil peritonitis model (**Figure 8**). The ovariectomized and castrated mice had sham surgery or sex organ removal at three weeks of age, preventing hormonal exposure at puberty. Mice were studied at 10 weeks of age.

We noted several interesting outcomes in this model. As expected, sham female mice demonstrated greater neutrophil influx than sham males, supporting female-dominant effects of IL-33. Importantly, the sham female group also showed greater neutrophilia than ovariectomized females, suggesting female sex hormones promote the inflammatory response. Mirroring this, sham males showed less neutrophil influx than castrated males, indicating male sex hormones diminish IL-33 effects. Most strikingly, removing male or female sex organs completely obviated sexual dimorphism. Ovariectomized females treated with IL-33 showed no significant differences in neutrophil infiltration than either sham or castrated males receiving IL-33. Likewise, castrated males responded to IL-33 similarly to sham or ovariectomized females. Thus, when measuring IL-33-induced neutrophil influx as an end point, female sex hormones appear to play an inflammatory role, while male hormones appear to repress the response. Moreover, sex organs were essential for sexual dimorphism, as their removal yielded statistically equal responses among the sexes.

In addition to neutrophil influx, we also measured plasma IL-13. Sham females exhibited 4-fold greater IL-13 production than sham males, supporting sexual dimorphism in the response. It is worth noting that sham males produced no detectable IL-13 upon challenge. More important and surprising was the finding that ovariectomy or castration had no significant effect on IL-13 production. Sham and ovariectomized females responded similarly to IL-33, as did sham and castrated males. Further, ovariectomized females still produced more IL-13 than sham males, and castrated males produced much less IL-13 than sham females. This lack of change in response to sex organ removal suggests IL-13 production is sexually dimorphic but independent of sex hormones.

## Discussion

Sexual dimorphism, defined as phenotypic differences between biological sexes, is notable in inflammatory and autoimmune diseases, which are more common in females than males (28). This is true for many allergic diseases, where adult females have a higher incidence of allergic asthma, allergic rhinitis, food allergy, atopic dermatitis, chronic urticaria, and mast cell activation disorders (29–31). These diseases share important inflammatory components, including a Th2-biased response that promotes IgE production, mast cell activation, and eosinophil recruitment (32, 33).

In addition to these canonical components of allergic disease, there is a growing understanding of a relatively new contributor: IL-33. This unusual cytokine is constitutively produced by structural cells, including endothelial and epithelial cells, where it is bound to chromatin until cell damage or inflammatory signals prompt its release and proteolytic processing to a mature form with high affinity for the ST2 receptor (34, 35). Polymorphisms in *Il1rl1*, which encodes ST2, are a risk factor for asthma [35]. IL-33 signaling promotes allergic inflammation, activating mast cells, ILC2, a subset of Th2 cells, eosinophils, neutrophils, and basophils (36, 37). This inflammatory role has led to multiple clinical trials targeting IL-33-ST2 signaling (38, 39).

How IL-33 contributes to the female-dominant traits in allergic disease is unclear. Mathä et al. showed that naïve female C57BL/6 mice have higher baseline levels of IL-33 in lung tissue than males and that intranasal (i.n.) challenge with IL-33 elicited greater eosinophil recruitment to the lungs in females (16). Aldossary et al. linked these effects to ILC2, showing that purified ILC2 from females secrete more IL-13 and IL-5 when challenged with IL-33 than male ILC2 (40). They also found greater i.n. responses to IL-33 among female BALB/c mice, as measured by eosinophil and ILC2 infiltration. Wang et al. reported similar results, showing increased airway inflammation in IL-33-challenged female mice (41). This group also found that testosterone suppressed IL-5, IL-9, and IL-13 production by human ILC2 cells stimulated with a combination of IL-2/25/33. Thus, there is strong support for female-dominant IL-33 responses among ILC2 and an indication that sex hormones can play a role.

Mast cells are IL-33 potent responders and prominent in allergic disease. Mackey et al. and Hox et al. found that female mast cells are more responsive to IgE-mediated anaphylaxis (42, 43). Given these papers and evidence that IL-33 function is sexually dimorphic among ILC2, we hypothesized that IL-33 effects on mast cells are female-dominant. Our results clearly show this *in vitro* and *in vivo*. Female dominance also extended to IL-33 effects on purified neutrophils but not eosinophils. Interestingly, sex differences in neutrophil biology and function are well documented, but similar data for eosinophils is not apparent in the literature (44). Our data, supported by the work of others, offers a collective view of IL-33 acting dominantly on female innate immune cells, but with lineage selectivity.

Several caveats accompany our results. Our *in vitro* studies included the variable of phenol red dye present in female culture media but not male media. Importantly, phenol red (present in RPMI at 15 μM) has estrogenic effects, binding estrogen receptors with a Kd of approximately 20 μM and inducing ER-dependent effects (17, 18). The inhibitory effects of phenol red were evident in our male BMMC cultures, which expanded poorly and responded weakly to IL-33 when the dye was present. Female BMMC cultured with or without phenol red responded comparably to IL-33, but cultures lacking phenol red yielded few cells. We suggest that this culture media variable is important because it more closely represents the *in vivo* environment. 17β-estradiol blood levels in reproductive-age females are approximately 0.4 nM, matching its 0.1-0.4 nM binding affinity for ERα and ERβ, respectively (45, 46). Thus, phenol red is present in culture media at a similar potency as 17β-estradiol in blood. In contrast, circulating 17β-estradiol levels in males are below 0.1 nM (46). Most importantly, our *in vitro* results were corroborated by two *in vivo* assays requiring mast cell responses to IL-33.

An important consideration is the possibility of differing mast cell numbers in male and female mice. We found no difference in peritoneal mast cells, the most likely mast cell population activated in our *in vivo* assays. One group found 1.5-fold greater mast cell density in the C57BL/6 mouse ileum, but human studies of the skin and intestine have found no sex differences in mast cell number (47–49). Therefore, it seems unlikely that the greater IL-33 response we found in females is due to differences in mast cell number.

We identified cellular mechanisms controlling the 4-day eosinophilic peritonitis model. Our data show that ILC2s are absolutely required for IL-33-mediated eosinophil recruitment, while mast cell depletion reduced eosinophil influx by more than half. We interpret this to mean that both ILC2 and mast cells are early IL-33 responders that promote eosinophil migration *in vivo*. Among the IL-33-responsive lineages, our data with eosinophils was curious. We found that ST2 must be expressed on eosinophils for their recruitment in the peritonitis model, and that IL-33 injection recruited more eosinophils in females than males. This could suggest that female eosinophils are more responsive to IL-33. However, male and female eosinophils cultured from bone marrow precursors showed identical IL-33-induced cytokine production. We tested eosinophil migration *in vitro* but were unable to induce migration towards IL-33 in Transwell assays. Our data are consistent with similar findings by Angulo et al. and Andreone et al, who showed that IL-33 does not directly induce eosinophil migration (25, 26). Importantly, ST2 deletion from eosinophils did not reduce eosinophil numbers in these mice. In fact, bone marrow eosinophil counts were significantly elevated in *Epx^Cre/+^;Il1rl1^fl/fl^* mice, though this difference was not noted in the spleen. We interpret data from the eosinophilic peritonitis model to mean that IL-33 signaling on eosinophils is necessary for their recruitment but (1) the actual migration signal is not IL-33 and (2) IL-33-ST2 function on eosinophils is not dimorphic We postulate that either the production of the eosinophil migration signal is sexually dimorphic and/or IL-33 selectively sensitizes female eosinophils to the chemotactic stimulus. This hypothesis agrees with our evidence that female mast cells produce more MCP-1 and MIP-1α than males (**Figure 1**) and similar findings from Aldossary et al. studying ILC2 cytokine secretion (40).

It was interesting to find no difference in surface ST2 or KIT expression among purified male and female mast cells or neutrophils, despite greater function in females. Mathä et al. found no variation in ST2 levels on male and female ILC2, while Aldossary et al. showed that among IL-33-challenged mice, female ILC2 expressed significantly more ST2 than males (16, 40). In our dose-response data from **Figure 1A**, we observed that female BMMC mounted a greater response to IL-33 without showing increased sensitivity, such as a lower EC_50_. Instead, we found that male and female mast cells have qualitative differences in ST2 signaling. Despite greater IL-33-induced ERK and p38 activation in male mast cells, these pathways appeared to be required for IL-33 function in both sexes. In contrast, females showed a 40-fold activation of JNK, a pathway not detected in males. More importantly, blocking JNK in males had no effect, while it greatly reduced IL-33-mediated cytokine production by female mast cells. Thus, our data point to the JNK pathway as a molecular mechanism supporting stronger IL-33 responses in female mast cells.

While IL-33-mediated MAPK activation is established, most papers point to p38 as the critical kinase regulating mast cell ST2 signaling (50). For example, resveratrol and berberine sulfate suppress IL-33-mediated mast cell cytokine production by inhibiting p38 but not JNK (51–53). Human mast cells have also been shown to respond to IL-33 in a p38-dependent manner (51). This study detected JNK activation but did not test its importance in cytokine release. In contrast, we previously found that lactic acid-mediated suppression of mast cell IL-33 responses correlated with loss of JNK but not p38 activation (54). Andrade et al. showed that suppressing either JNK or p38 diminished IL-33-mediated TNF production by BMMCs (50). Hence, there is support for JNK-mediated responses to IL-33 in mast cells. We previously found that IL-33-induced AP-1 DNA binding activity correlated with cytokine production, but that study did not test the functional importance of JNK or AP-1 (55). Further work is needed to determine how JNK amplifies IL-33 signaling in female mast cells and why this pathway is not active in male mast cells.

Sexual dimorphism can most often be traced to the effects of sex hormones or gene copy number, as females carry two X chromosomes with incomplete X-inactivation (56). Sex hormone effects are a likely explanation for our data, based on literature showing that estrogen promotes mast cell function, while androgens can be inhibitory. For example, Mackey et al. found that perinatal androgen exposure limits the mast cell response (57). Others have found that estrogen promotes mast cell responses, although IL-33-mediated stimulation has not been examined (58–62). Using ovariectomized or castrated mice, we found that male and female sex hormones have opposing effects on IL-33-induced neutrophil recruitment but surprisingly, no effect on plasma IL-13. This dichotomy indicates distinct controls over these two measures of inflammation. We postulate that sex hormones impact neutrophil recruitment but X-chromosome gene dose effect is more important for differences in IL-13 secretion. While mast cell-derived TNF-α is necessary for neutrophil recruitment in the 4-hour model, we find its levels to be below detection in plasma (not shown) (63). IL-13 has potent inflammatory effects, including T cell recruitment, goblet cell metaplasia, and fibrotic remodeling of the airways and skin (64). It is striking that male mice showed no IL-33-induced IL-13 production in the 4-day IL-33 challenge model, exhibiting not just quantitative but qualitative differences from matched females. The lack of sex hormone effects suggests another mechanism, possibly a gene dose effect from the X chromosome, controls IL-13 production.

An important caveat for our studies using sex organ removal is the possibility of changes in tissue mast cells or circulating neutrophils, which we did not quantify. There is considerable literature surrounding these effects. Ovariectomy had no effect on intestinal mast cell numbers, although it increased mast cells in the bone marrow (65, 66). Kuhn et al. showed that ovary removal had no effect on peripheral neutrophils (67). Therefore, a systemic loss of mast cells or neutrophils seems to be an unlikely explanation for reduced neutrophil infiltration in ovariectomized mice. Orchiectomy has been shown to have no effect on peripheral neutrophils or skin mast cell numbers, but did increase mast cells in the prostate (68–71). These studies suggest that increased neutrophilic peritonitis in castrated males is not likely caused by a significant increase in neutrophils or mast cells. On balance, our data support an important role for sex organs in sexually dimorphic IL-33 responses *in vivo*, but the precise role of sex hormones is unclear from our current studies.

Collectively, we show that IL-33 responses are stronger in female mast cells and neutrophils, but not eosinophils. These data add to previous work showing female-dominant IL-33 effects on ILC2 (16, 40, 41). Previous studies also found stronger IgE-induced mast cell responses in females (42, 43). Our data extend this observation to IL-33 and should prompt further study of the many known mast cell-activating stimuli. Importantly, our work provides a molecular mechanism for female-specific effects. ST2-mediated JNK signaling is absent in male mast cells and appears to selectively amplify the female responses. While male mast cells show stronger activation of ERK and p38, inhibiting these pathways suppressed IL-33 function in both sexes, unlike JNK targeting. Future work needs to delineate how JNK signaling is selectively employed and if sex hormones contribute to this signaling cascade. In this regard, there is considerable literature showing variable effects of estrogen on the JNK-AP-1 pathway. Sun et al. showed that JNK1 and ERα can interact at some promoters and function as coregulators in breast cancer cells (72). Mutant ERα can also induce JNK-AP-1 function in breast cancer cells (73). Extending these lines of research into allergic disease could provide fundamental insight into diseases that are more common and more severe in females.

## Data Availability

The original contributions presented in the study are included in the article/**Supplementary Material**. Further inquiries can be directed to the corresponding author.
