# Supplementary figures and images for: IL-33 induces stronger responses in female mast cells and neutrophils: a role for the JNK pathway
Source: Front Immunol. 2026 Jul 7;17:1813530. doi: 10.3389/fimmu.2026.1813530 (PMC13384896; doi:10.3389/fimmu.2026.1813530)

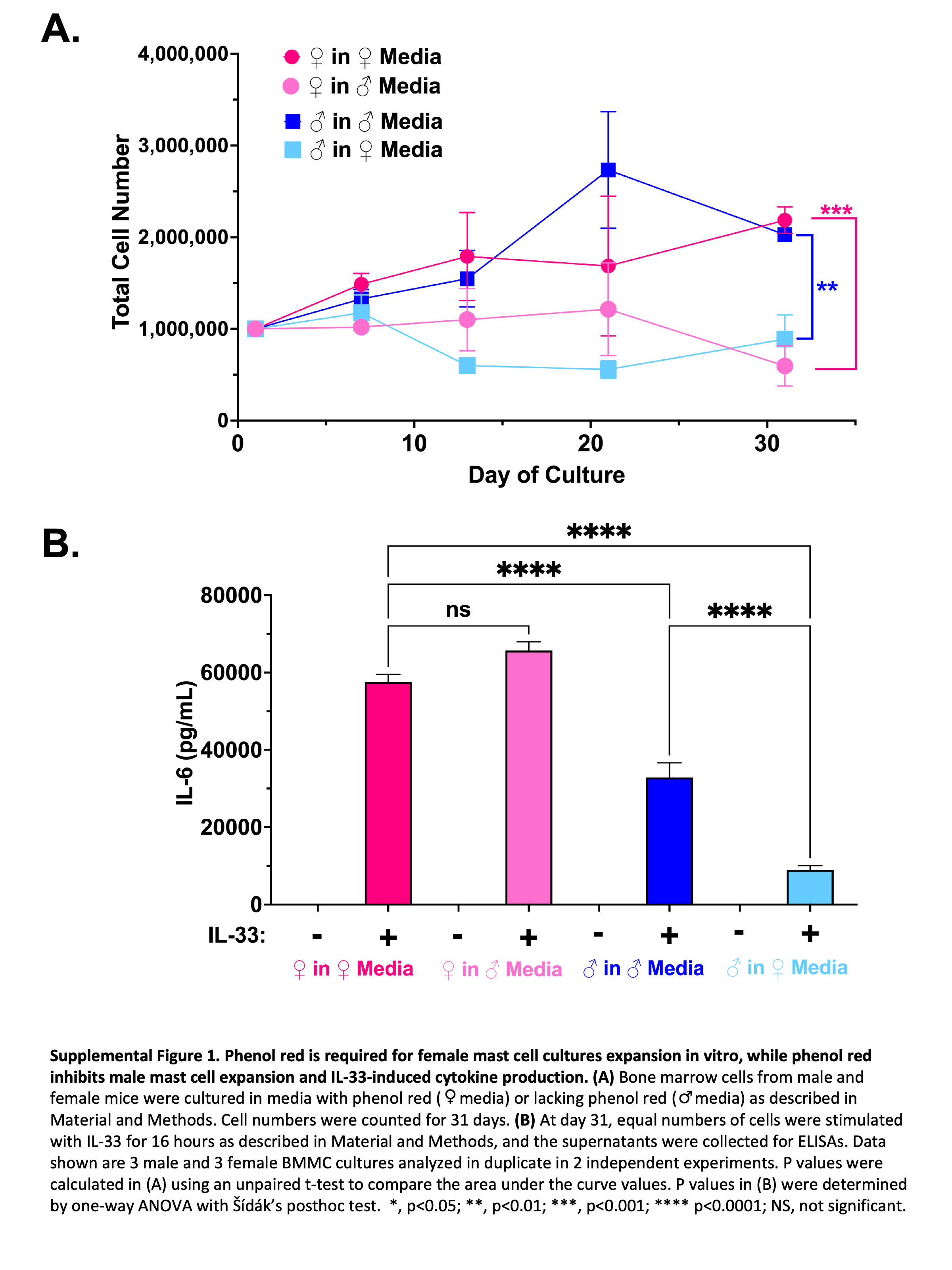

Supplement: Supplementary file 1 [file Image1.tiff]

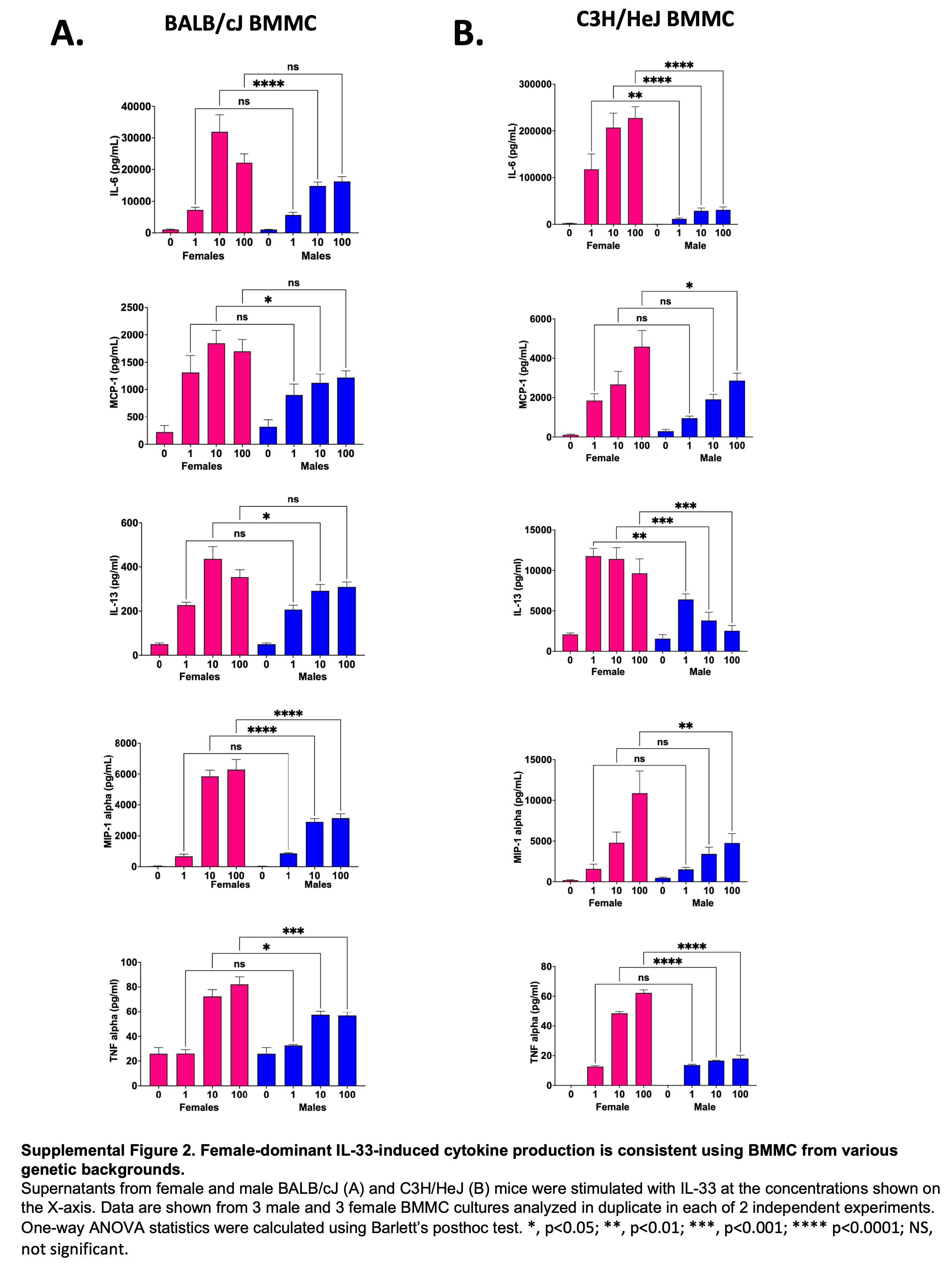

Supplement: Supplementary file 2 [file Image2.tiff]

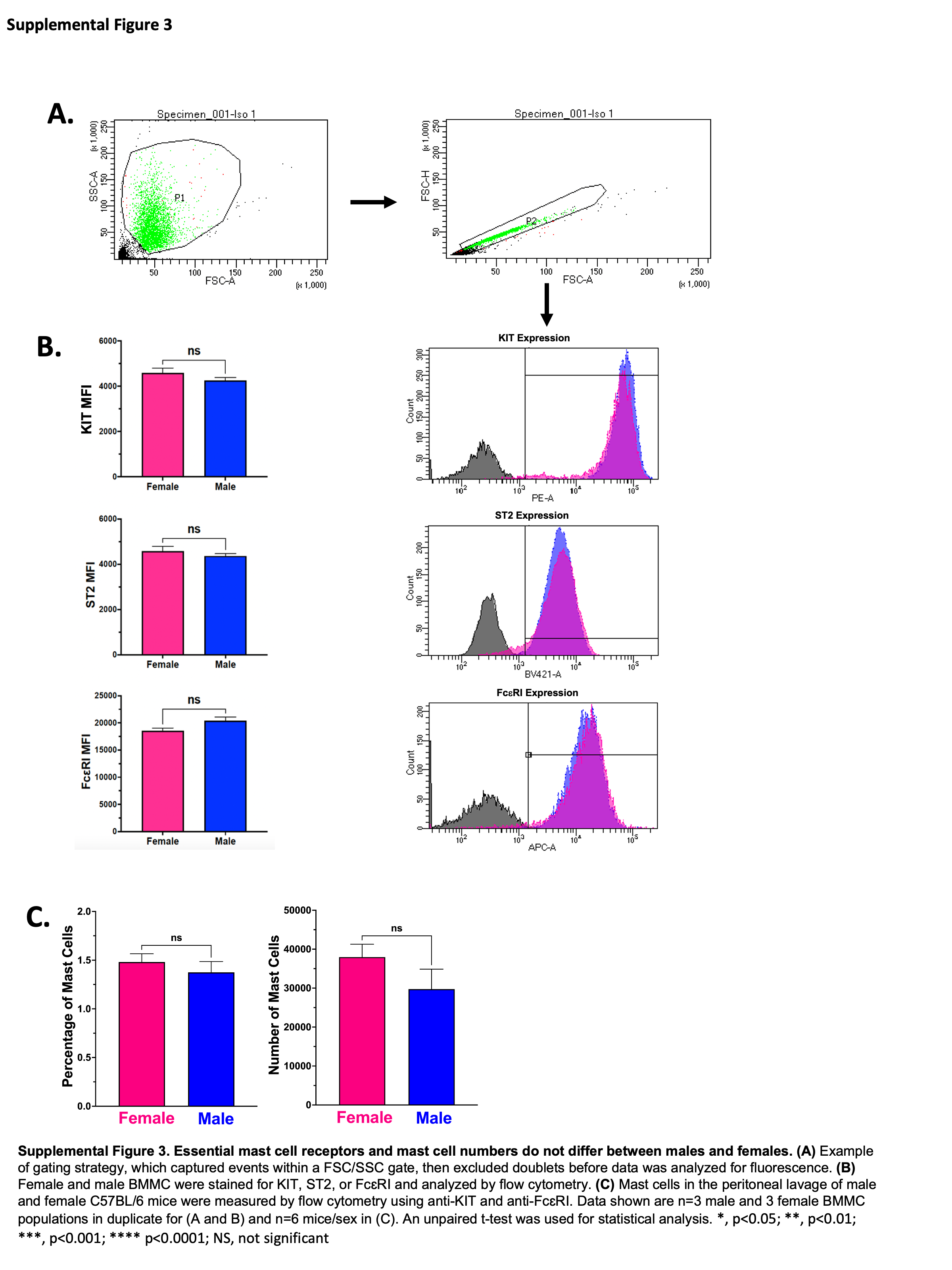

Supplement: Supplementary file 3 [file Image3.tiff]

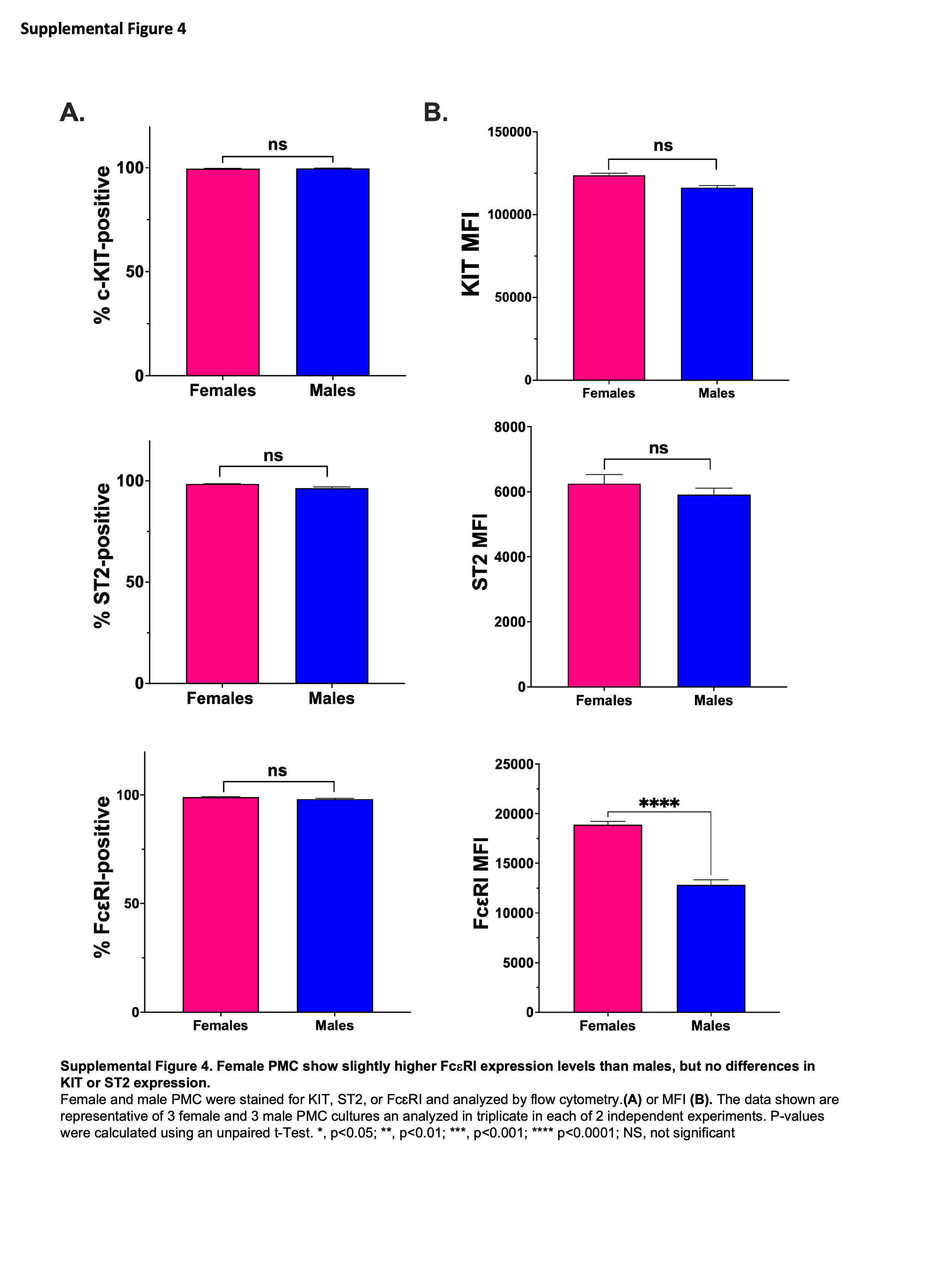

Supplement: Supplementary file 4 [file Image4.tiff]

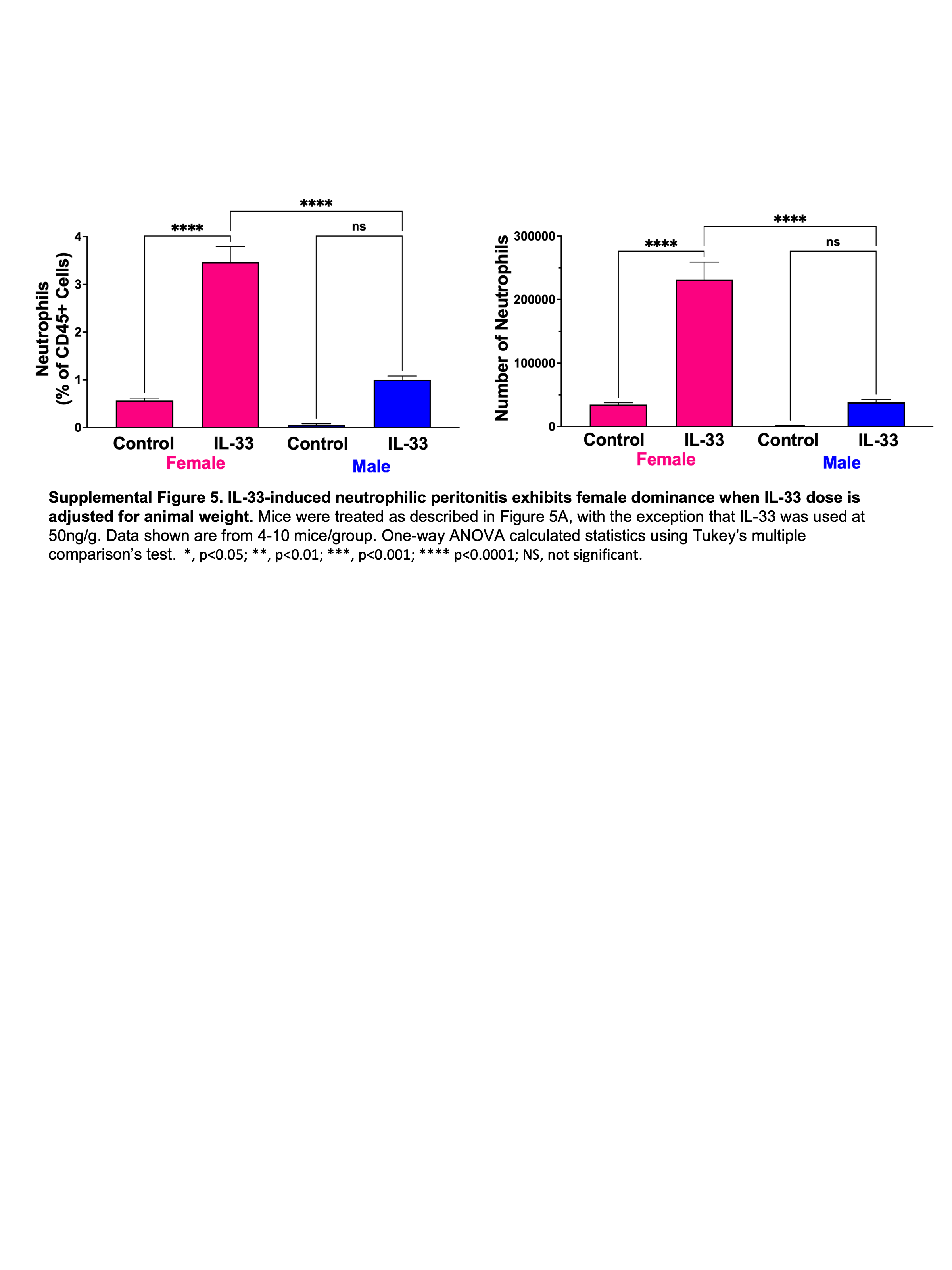

Supplement: Supplementary file 5 [file Image5.tiff]

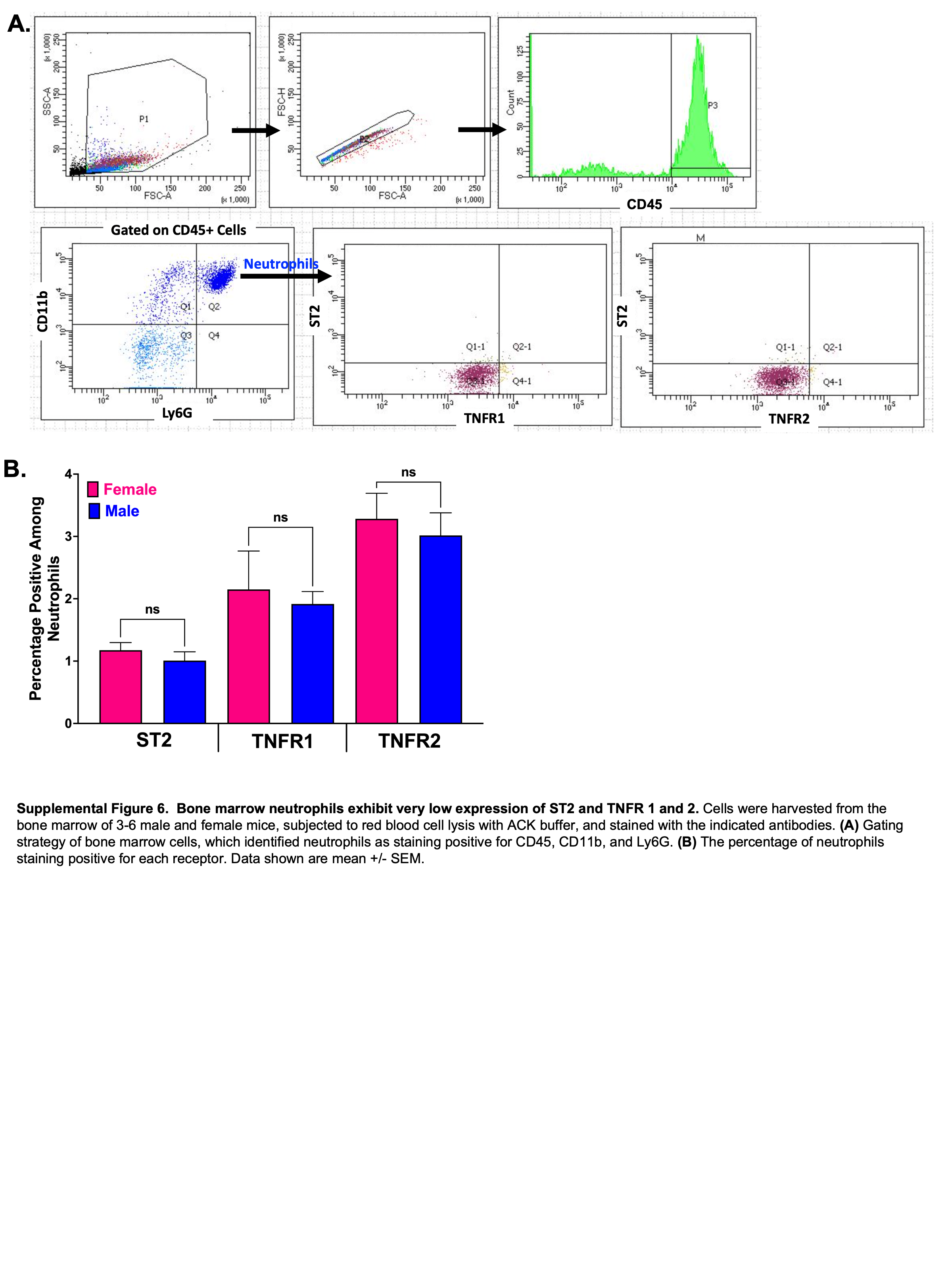

Supplement: Supplementary file 6 [file Image6.tiff]

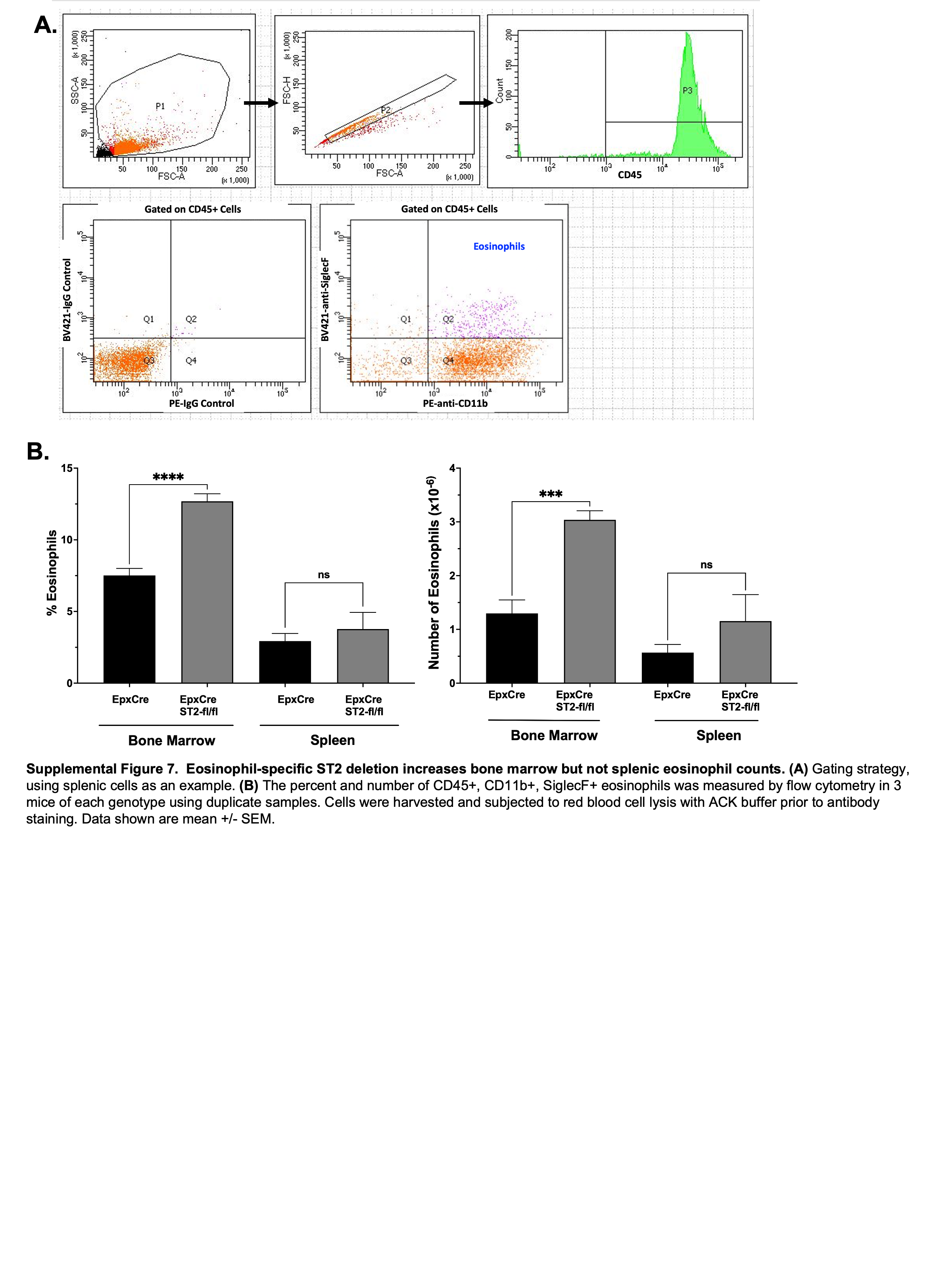

Supplement: Supplementary file 7 [file Image7.tiff]
